# Antioxidant Power of Brown Algae: *Ascophyllum nodosum* and *Fucus vesiculosus* Extracts Mitigate Oxidative Stress In Vitro and In Vivo

**DOI:** 10.3390/md23080322

**Published:** 2025-08-06

**Authors:** Lea Karlsberger, Georg Sandner, Lenka Molčanová, Tomáš Rýpar, Stéphanie Ladirat, Julian Weghuber

**Affiliations:** 1 Center of Excellence Food Technology and Nutrition, University of Applied Sciences Upper Austria, Stelzhamerstraße 23, 4600 Wels, Austria; lea.karlsberger@fh-wels.at; 2 FFoQSI GmbH, Austrian Competence Centre for Feed and Food Quality, Safety and Innovation, Technopark 1D, 3430 Tulln, Austria; georg.sandner@ffoqsi.at; 3 Department of Natural Drugs, Faculty of Pharmacy, Masaryk University, Palackého Třída 1946/1, 612 00 Brno, Czech Republic; molcanoval@pharm.muni.cz; 4 Department of Chemistry and Biochemistry, Mendel University in Brno, Zemědělská 1665/1, 613 00 Brno, Czech Republic; tomas.rypar@gnj.cz; 5 NUQO S.A.S, 13 Rue d’Albigny, 74000 Annecy, France; ladirat.stephanie@nuqo.eu

**Keywords:** brown algae, oxidative stress, *C. elegans*, ROS, antioxidant, phlorotannins, *Ascophyllum nodosum*, *Fucus vesiculosus*, gene expression

## Abstract

Brown algae such as *Ascophyllum nodosum* (AN) and *Fucus vesiculosus* (FV) are gaining considerable attention as functional feed additives due to their health-beneficial properties. This study evaluated the antioxidant potential of AN and FV extracts in intestinal epithelial cells and the in vivo model *Caenorhabditis elegans* (*C. elegans*). Aqueous AN and FV extracts were characterized for total phenolic content (TPC), antioxidant capacity (TEAC, FRAP), and phlorotannin composition using LC-HRMS/MS. Antioxidant effects were assessed in vitro, measuring AAPH-induced ROS production in Caco-2 and IPEC-J2 cells via H_2_DCF-DA, and in vivo, evaluating the effects of paraquat-induced oxidative stress and AN or FV treatment on worm motility, GST-4::GFP reporter expression, and gene expression in *C. elegans*. FV exhibited higher total phenolic content, antioxidant capacity (TEAC, FRAP), and a broader phlorotannin profile (degree of polymerization [DP] 2–9) than AN (DP 2–7), as determined by LC-HRMS/MS. Both extracts attenuated AAPH-induced oxidative stress in epithelial cells, with FV showing greater efficacy. In *C. elegans*, pre-treatment with AN and FV significantly mitigated a paraquat-induced motility decline by 22% and 11%, respectively, compared to PQ-stressed controls. Under unstressed conditions, both extracts enhanced nematode healthspan, with significant effects observed at 400 µg/g for AN and starting at 100 µg/g for FV. Gene expression analysis indicated that both extracts modulated antioxidant pathways in unstressed worms. Under oxidative stress, pre-treatment with AN and FV significantly reduced GST-4::GFP expression. In the nematode, AN was more protective under acute stress, whereas FV better supported physiological function in the absence of stressors. These findings demonstrate that AN and FV counteract oxidative stress in intestinal epithelial cells and in *C. elegans*, highlighting their potential as stress-reducing agents in animal feed.

## 1. Introduction

In recent years, restrictions on antibiotic growth promoters (AGPs) in livestock production and growing consumer concerns have accelerated the search for natural, plant-based feed additives improving animal health and productivity in a sustainable manner [1,2]. The livestock industry faces increasing regulatory pressure and market demand for more environmentally friendly and residue-free alternatives to synthetic additives, driving interest in natural compounds that support animal resilience, immunity, and performance [3,4]. In this regard, marine macroalgae, particularly brown seaweeds, have gained considerable attention due to their nutritional value and rich profile of bioactive compounds [5,6].

Brown algae are a diverse group of macroalgae characterized by diverse bioactive molecules, including polysaccharides (e.g., alginic acid, laminarin, fucans), polyunsaturated fatty acids, vitamins, minerals, peptides, and polyphenolic compounds known as phlorotannins [7]. These constituents exhibit a wide range of biological activities including antioxidant, anti-inflammatory, antimicrobial, and immunomodulatory effects [8,9]. Accordingly, brown algae have been reported as dietary supplements in monogastric and ruminant animals, with evidence suggesting improved immunity, stress resistance, and overall health [10,11]. Among these, *Ascophyllum nodosum* and *Fucus vesiculosus*, both abundant along the North Atlantic coast, are notable for their high phlorotannin content and ongoing use in animal nutrition [12].

The biological relevance of brown algae is largely attributed to their antioxidant potential, particularly their ability to counteract reactive oxygen species (ROS). ROS, such as superoxide anion (O_2_^−^), hydrogen peroxide (H_2_O_2_), and hydroxyl radicals (•OH), are produced as natural byproducts of cellular metabolism, especially during mitochondrial oxidative phosphorylation [13]. While low levels of ROS serve as signaling molecules involved in various physiological processes, excessive ROS production or impaired antioxidant defenses can lead to oxidative stress, resulting in severe damage to proteins, lipids, and DNA [14] and various diseases [15,16,17,18] in animals.

The gastrointestinal tract of livestock is particularly susceptible to oxidative stress due to the high metabolic activity of intestinal epithelial cells and the presence of microbial metabolites and dietary antigens [19]. In this context, dietary antioxidants play a critical role in maintaining intestinal integrity, modulating immune responses, and enhancing overall animal health and productivity [20]. Hence, natural antioxidants from seaweeds, especially those with high phlorotannin content, may offer a dual benefit by improving oxidative balance and exerting antimicrobial effects against gut pathogens [21].

Although a number of in vitro studies have reported antioxidant activity in brown algae extracts [22,23,24], there remains a significant gap in in vivo research, especially under conditions relevant to animal nutrition. This lack of in vivo evidence constrains the comprehensive assessment of their efficacy, safety, and practical application as functional feed additives in livestock production. However, in vivo trials remain costly, time-consuming, and subject to ethical considerations, necessitating the use of alternative models to bridge the knowledge gap [25].

In this context, the nematode *C. elegans* emerged as a robust alternative in vivo model for assessing the antioxidant potential of natural compounds. With a fully sequenced genome, conserved signaling pathways, and a short lifespan, *C. elegans* provides an ethical and cost-effective platform for higher-throughput screening of bioactive compounds [26]. Furthermore, the transparent body allows for direct observations of oxidative stress markers and age-related phenotypes, while transgenic strains expressing oxidative stress reporters enable mechanistic investigations at the molecular level [27].

Research specifically investigating the effects of brown algae, such as *Ascophyllum nodosum* and *Fucus vesiculosus*, in *C. elegans* models remains limited [28,29]. The aim of this study was to investigate the antioxidant activity and biological effects of *Ascophyllum nodosum* (AN) and *Fucus vesiculosus* (FV) extracts in both in vitro (Caco-2 and IPEC-J2 cells) and in vivo (*C. elegans*) models. The study specifically seeks to evaluate their efficacy in mitigating oxidative stress, assess their impact on organismal healthspan, and elucidate the underlying molecular mechanisms, including the modulation of antioxidant gene expression and stress-responsive pathways. This study combines phenolic profiling with functional and molecular analyses to support a deeper understanding of the bioactivity, bioavailability, and specific effects of antioxidants from brown algae.

## 2. Results

### 2.1. Contrasting Phenolic, Antioxidant, and Polysaccharide Contents in AN and FV

The antioxidant and polysaccharide contents of AN and FV were evaluated through photometric assays, quantifying the total phenolic content (TPC), Trolox equivalent antioxidant capacity (TEAC), ferric reducing antioxidant power (FRAP), and total polysaccharide content (TPSC). Aqueous extracts of AN and FV were analyzed photometrically, with phloroglucinol used as the standard for TPC, Trolox as the reference for TEAC and FRAP, and D-glucose as the standard for TPSC. All measurements were normalized to the dry weight of the algal material, using standard calibration curves for each assay.

**Figure 1:**
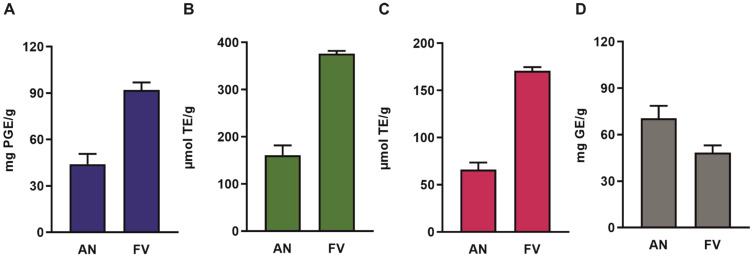
TPC, TPSC, and antioxidant capacity of AN and FV extract. (**A**) TPC expressed as mg phloroglucinol equivalents (PGE) per g dry algae, (**B**) TEAC and (**C**) FRAP expressed as µmol Trolox equivalents (TE) per g dry algae, and (**D**) TPSC expressed as mg glucose equivalents (GE) per g dry algae were measured using colorimetric assays. Data represent mean ± SD of three replicates from three independent extracts.

As shown in Figure 1, FV exhibited approximately 3-fold higher TPC, TEAC, and FRAP values than AN. These results suggest a higher concentration of phenolic compounds and stronger antioxidant potential in FV. In contrast, AN exhibited a markedly greater polysaccharide content, reaching 71 mg glucose equivalents (GE)/g DW, compared to 48 mg GE/g DW in FV.

### 2.2. Tentative Identification of Phlorotannins via LC-HRMS/MS Analysis

Phlorotannins (PTs) in aqueous extracts of AN and FV were tentatively identified using LC-HRMS/MS in negative electrospray ionization mode. Compound identification was based on accurate mass measurement, isotope pattern, retention time (RT), and detection of characteristic fragment ions or neutral losses in MS^2^ spectra. The degree of polymerization (DP) was estimated by the number of phloroglucinol units present in the chemical formula.

Table 1 summarizes ten PTs detected in the AN extract. The calculated molecular weights ranged from 250.05 to 870.13 Da, corresponding to PTs with a DP from 2 to 7. All compounds were detected as deprotonated molecular ions [M–H]^−^, and no MS^2^ spectra were acquired for compounds PT1–PT9. One compound (PT10) triggered the data-dependent acquisition (DDA) of MS^2^ spectra and was further supported by fragment ion information.

Table 2 presents 33 tentatively identified PTs in the FV extract, showing a broader structural diversity with molecular weights ranging from 250.05 to 1118.16 Da and DPs from 2 to 9. Several isomers were observed based on identical molecular formulas but different retention times. MS^2^ fragmentation was successfully triggered for selected compounds (e.g., PT3, PT4, PT7, PT11, PT18, PT25, PT27, PT29, PT30), which provided additional structural support. Notably, higher DPs (≥8) such as PT29–PT33 were detected exclusively in the FV extract.

All detected PTs were annotated based on their accurate masses within a tolerance of 5 × 10^−6^ (relative mass error) and retention time reproducibility across triplicate runs. Neutral losses characteristic for phlorotannin structures were used for confirmation during MS^2^ interpretation, when available. Identification was tentative and based on literature-reported compositions and an in silico-generated database of potential PTs.

### 2.3. AN and FV Reduce ROS Production Under Stress Conditions in Intestinal Epithelial Cells

To evaluate the antioxidant efficacy of AN and FV extracts under oxidative stress, intracellular ROS levels in human Caco-2 and porcine IPEC-J2 intestinal epithelial cells were measured following treatment with 100, 200, or 400 µg/g of each extract. Prior to assessing antioxidant activity, potential cytotoxic effects of the extracts were examined. Neither AN nor FV exhibited negative effects on cell viability at the tested concentrations (Supplementary Figure S1).

Oxidative stress was induced using 200 µM 2,2′-azobis(2-amidinopropane) dihydrochloride (AAPH), and quercetin (QUE) at a concentration of 20 µM served as a positive control.

**Figure 2:**
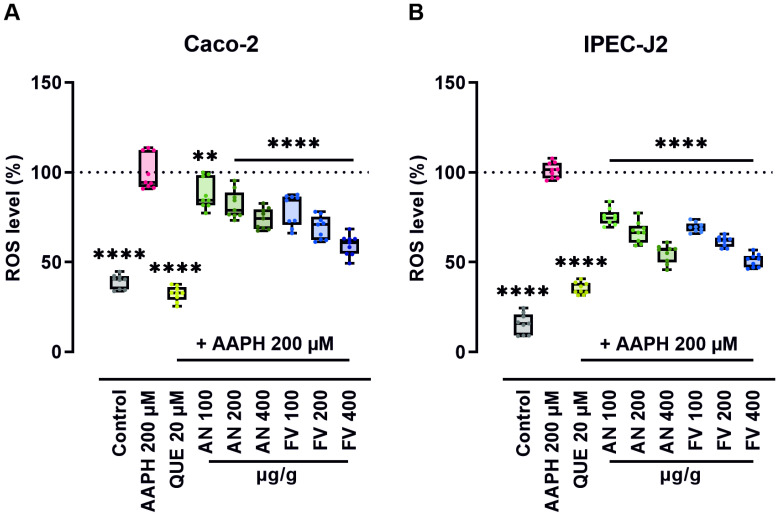
AN and FV reduce intracellular ROS production in Caco-2 and IPEC-J2 cells under AAPH-induced stress. (**A**) Human Caco-2 and (**B**) porcine IPEC-J2 cells were seeded into 96-well plates at a density of 5 × 10^4^ or 2 × 10^4^ cells per well, respectively, and grown overnight. After a 20 min co-treatment with 50 µM H_2_DCF-DA and AN or FV at 100, 200, and 400 µg/g, or 20 µM QUE as a positive antioxidant control, oxidative stress was induced with 200 µM AAPH. The fluorescence intensity (FI) of DCF was monitored for 90 min. The ROS level over time was summarized by calculation of the area under the curve (AUC) and normalization to the AAPH-treated stress control. Statistical analysis was performed using one-way ANOVA with Dunnett’s multiple comparison test. Data are presented as box plots showing the median, interquartile range (IQR), whiskers (min/max values), and individual data points from three independent experiments. Significant *p* values are indicated as ** (*p* ≤ 0.01), **** (*p* ≤ 0.0001).

As shown in Figure 2A,B, both AN and FV extracts significantly reduced AAPH-induced ROS levels in a dose-dependent manner in both cell lines. In Caco-2 cells, AN treatment (100–400 µg/g) reduced ROS levels by 12–26% (*p* ≤ 0.01 to *p* ≤ 0.0001), whereas FV achieved a more pronounced reduction of 21–41% within the same concentration range (*p* ≤ 0.0001). Similarly, in IPEC-J2 cells, AN decreased ROS levels by 25–45%, while FV elicited a stronger response, reducing ROS by 30–49% (*p* ≤ 0.0001). Across all tested concentrations, FV consistently exhibited greater ROS-scavenging activity compared to AN in both cell lines.

### 2.4. AN and FV Improve Worm Motility Under Stressed and Unstressed Conditions

We further investigated the effects of AN and FV on oxidative stress using the in vivo model *C. elegans*. As an indicator of nematode healthspan, motility was assessed, since it is closely associated with overall vitality and functional aging [30].

Prior to assessing oxidative stress responses, potential adverse effects of the extracts were examined. Neither AN nor FV exhibited toxicity or induced any negative impact on worm viability across the tested concentrations (Supplementary Figure S1), indicating that both extracts were well-tolerated under the chosen experimental conditions.

For stressed conditions, wild-type nematodes were pre-treated for 3 days with either AN or FV. Worm activity was recorded in 96-well plates after oxidative stress induction with 50 mM paraquat (PQ).

**Figure 3:**
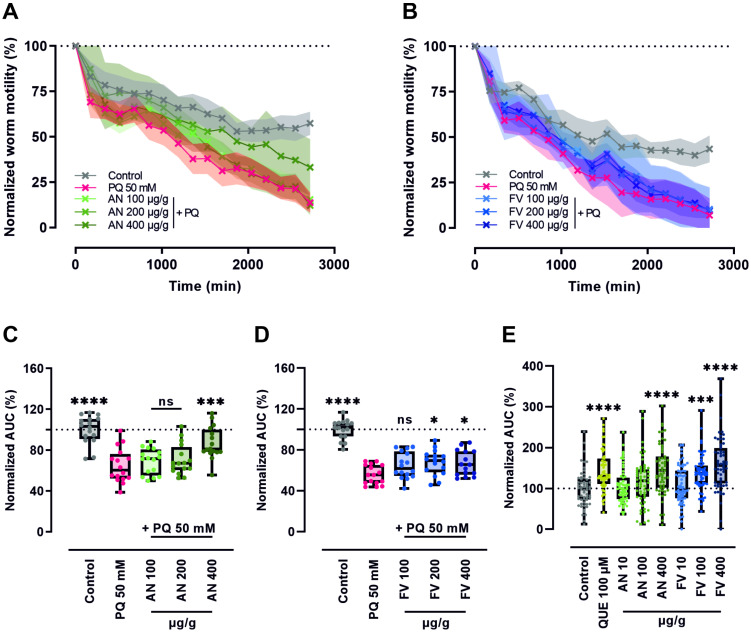
AN and FV improve worm motility under stressed and unstressed conditions. Age-synchronized wild-type *C. elegans* (strain N2) were incubated for 3 days on NGM agar supplemented with 100, 200, and 400 µg/g AN or FV. Worms (~80 per well) were then transferred to 96-well plates and exposed to 50 mM PQ to induce oxidative stress. Motility was monitored for 48 h using the WMicrotracker ONE system and normalized to baseline activity recorded 60 min prior to PQ exposure. The time course of worm motility following pre-treatment with (**A**) AN and (**B**) FV is presented as mean (line) ± SD (shaded area). (**C**,**D**) Overall worm motility was summarized by calculating the AUC over 48 h and normalization to the unstressed control. (**E**) For unstressed conditions, adult SS104 nematodes were treated with AN or FV (10, 100, and 400 µg/g), and single worms were individually transferred into wells of a 384-well flat bottom plate. Motility was tracked over 28 days. QUE (100 µM) was used as a positive antioxidant control. Worm motility was summarized by the calculation of the AUC over 28 days and normalization to the untreated control. Statistical analysis was performed using one-way ANOVA followed by Dunnett’s multiple comparison test. Data are shown as box plots indicating the median, interquartile range (IQR), whiskers (min/max values), and individual data points from two independent experiments with eight technical replicates per group for stressed conditions and three independent experiments with 20 individual worms per group for unstressed conditions. Significant *p* values are indicated as * (*p* ≤ 0.05), *** (*p* ≤ 0.001), **** (*p* ≤ 0.0001), or ns (not significant).

As shown in Figure 3A,B, PQ exposure resulted in a pronounced and progressive reduction in worm motility compared to the unstressed controls. Pre-treatment with higher concentrations of both AN and FV attenuated the PQ-induced decline in motility, suggesting a protective effect.

To quantitatively compare the impact of different treatments, we calculated the AUC over a 48 h period and normalized the values to the unstressed control (Figure 3C,D). PQ exposure markedly impaired motility, reducing mean AUC values to 65% and 56% of control levels in the respective datasets (both *p* ≤ 0.0001). Pre-treatment with AN at 400 µg/g alleviated this decline (Figure 3A), significantly increasing AUC by 22% compared to PQ-stressed controls (Figure 3C, *p* ≤ 0.001). FV pre-treatment elicited a dose-dependent improvement in motility (Figure 3B) with significant effects observed at 200 and 400 µg/g. At these concentrations, AUC values increased by 12% and 11%, respectively (*p* < 0.1), relative to the stress control (Figure 3D). These results indicate protective effects of both AN and FV against oxidative stress in *C. elegans*, with AN showing a more robust response at higher doses.

To further evaluate the potential healthspan-promoting effects of brown algae extracts under unstressed conditions, locomotion activity of the temperature-sensitive SS104 mutant strain was monitored. Nematodes were grown in liquid medium until adulthood followed by treatment with AN, FV, or QUE as a positive antioxidant control. The activity of single worms was tracked in a 384-well flat bottom plate for 28 days.

Worm motility, expressed as area under the curve (AUC) and normalized to untreated controls, is shown in Figure 3E. AN treatment at 400 µg/g significantly increased AUC by 46% (*p* ≤ 0.0001), indicating enhanced vitality over time. FV exhibited dose-dependent effects, with 100 µg/g increasing AUC by 38% (*p* ≤ 0.001) and 400 µg/g increasing AUC by 60% (*p* ≤ 0.0001), suggesting a stronger and earlier onset of healthspan-supporting activity compared to AN.

### 2.5. AN and FV Modulate Antioxidant Gene Expression in C. elegans Under Unstressed Conditions

Our findings indicate that AN and FV exert protective effects against oxidative stress in *C. elegans*. To elucidate the underlying transcriptional mechanisms, we quantified the relative mRNA expression of 14 key stress response genes via RT-qPCR. We included the central transcription factors *daf-2*, *daf-16*, *skn-1*, and *pmk-1* along with their major downstream targets *gcs-1*, *gpx-5*, *gst-4*, *mtl-1*, *prdx-2*, *sod-3*, and *sod-4*.

We assessed the impact of AN and FV on gene expression under both unstressed and PQ-induced oxidative stress conditions. Age-synchronized wild-type worms were treated with 100 or 400 µg/g of AN or FV in NGM agar for 48 h, followed by a 24 h exposure to either control or PQ-supplemented (20 mM) plates.

**Figure 4:**
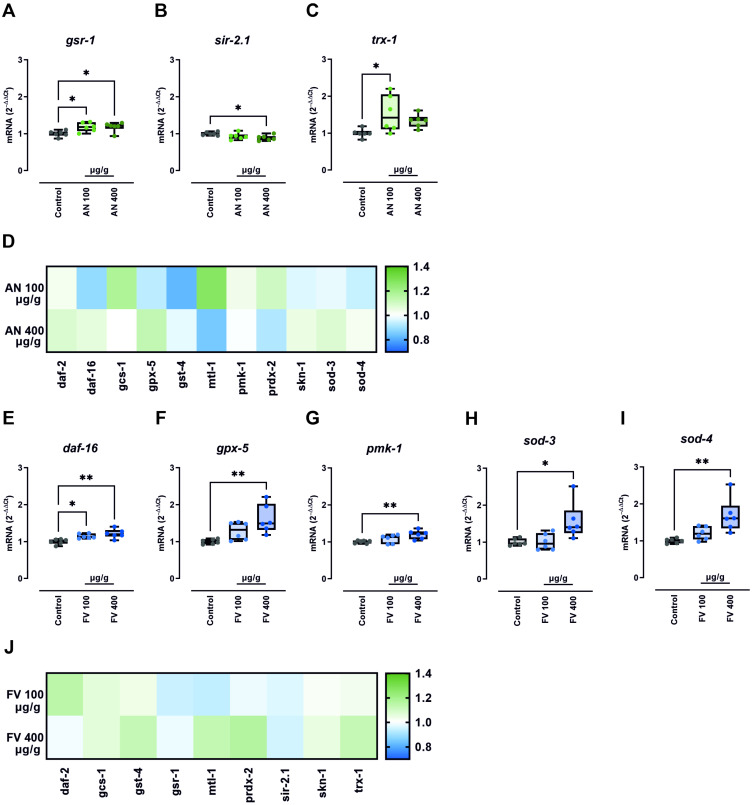
AN and FV modulate antioxidant gene expression in unstressed *C. elegans*. Age-synchronized wild-type nematodes were incubated on NGM agar containing 100 or 400 µg/g of AN or FV for 48 h. Nematodes were transferred to control plates and incubated for additional 24 h. Gene expression was quantitated by RT-qPCR and analyzed using the 2^−ΔΔCt^ method. Differences compared to untreated controls were evaluated by one-way ANOVA with Dunnett’s multiple comparison test. Significant changes in mRNA expression were observed for (**A**) *gsr-1*, (**B**) *sir-2.1*, (**C**) *trx-1*, (**E**) *daf-16*, (**F**) *gpx-5*, (**G**) mitogen-activated protein kinase *pmk-1*, (**H**) *sod-3*, and (**I**) *sod-4*. Data are presented as box plots showing the median, interquartile range (IQR), whiskers (min/max values), and individual data points from two independent experiments with three technical replicates per condition. Non-significant changes in gene expression following (**D**) AN and (**J**) FV treatment are summarized in respective heatmaps with data shown as mean values. Significant *p* values are indicated as * *p* ≤ 0.05, ** *p* ≤ 0.01.

Under unstressed conditions, 100 µg/g AN significantly upregulated the genes *gsr-1* and *trx-1* (Figure 4A,C) with fold changes of 1.2 and 1.5, respectively. At the higher concentration of 400 µg/g, AN also significantly increased *gsr-1* expression (1.2-fold; Figure 4A) and slightly downregulated *sir-2.1* (0.9-fold; Figure 4B). Similarly, FV treatment induced dose-dependent transcriptional changes. FV at 100 µg/g significantly elevated *daf-16* expression (1.2-fold; Figure 4E). whereas 400 µg/g FV further upregulated *daf-16*, *gpx-5*, *pmk-1*, *sod-3*, and *sod-4* (Figure 4E–J), with fold changes of 1.2, 1.6, 1.2, 1.6, and 1.7, respectively. Other tested genes were not found to be significantly changed and are summarized in the heatmaps (Figure 4D,H).

These findings indicate that AN and FV moderately activate antioxidant and stress response pathways in *C. elegans* under unstressed conditions.

Exposure to 20 mM PQ for 24 h significantly increased the expression of most stress response genes, reflecting robust activation of the antioxidant defense system. Interestingly, pre-treatment with AN or FV did not significantly modify the PQ-induced gene expression profiles. The extracts neither enhanced nor suppressed the transcriptional response triggered by PQ (Supplementary Figure S2).

### 2.6. AN and FV Reduce the Stress-Induced GST-4 Expression in Transgenic GFP Reporter Strains

**Figure 5:**
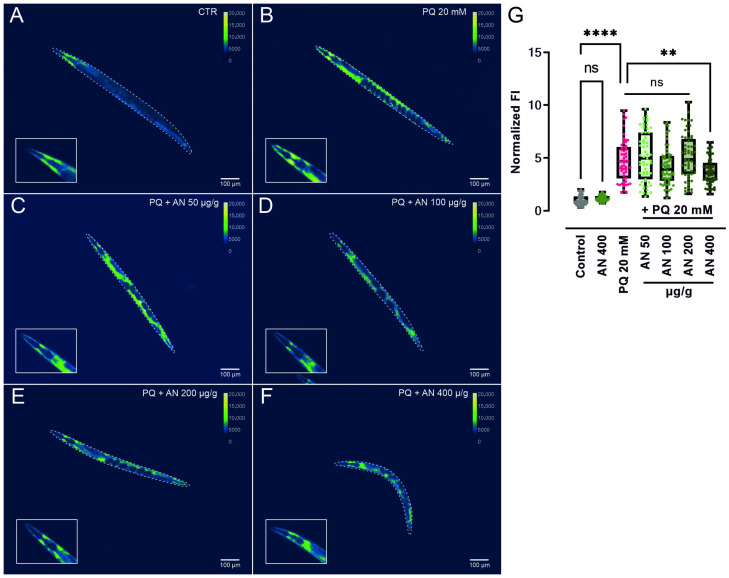
Expression of GST-4 is downregulated by AN in transgenic GFP reporter strains under oxidative stress. Age-synchronized VP596 [*gst-4*::GFP] nematodes were incubated on NGM agar containing (**A**,**B**) no treatment, (**C**) 50 µg/g, (**D**) 100 µg/g, (**E**) 200 µg/g, and (**F**) 400 µg/g AN for 2 days. For oxidative stress induction, L4 nematodes were incubated on NGM agar containing 20 mM of PQ for 24 h. Unstressed control worms were incubated on NGM for 24 h. Representative images of fluorescent protein expression were taken. (**G**) Mean FI of GST-4::GFP was quantified and normalized to the control. Differences from control and PQ treatment were analyzed using one-way ANOVA with Tukey’s multiple comparison test. Data are presented as box plots showing the median, interquartile range (IQR), whiskers (min/max values), and individual data points from three independent experiments with 20 individual worms per condition. Significant *p* values are indicated as ** (*p* ≤ 0.01), **** (*p* ≤ 0.0001) or ns (not significant).

**Figure 6:**
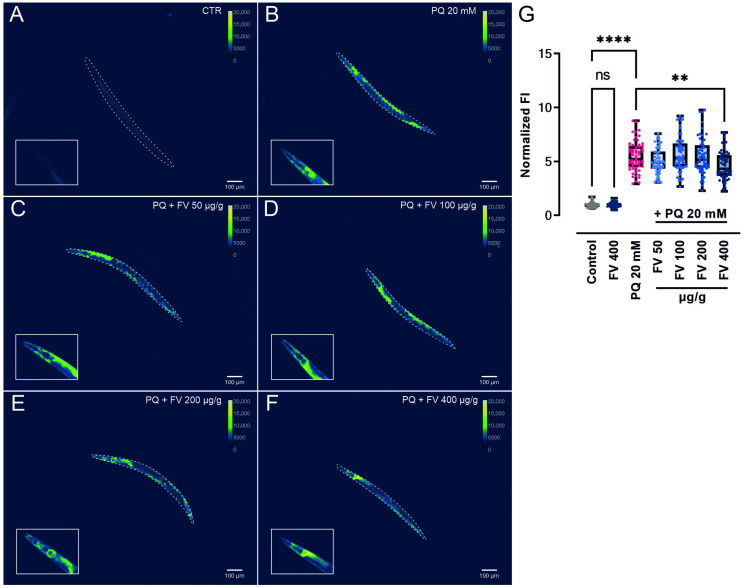
Expression of GST-4 is downregulated by FV in transgenic GFP reporter strains under oxidative stress. Age-synchronized VP596 [*gst-4*::GFP] nematodes were incubated on NGM agar containing (**A**,**B**) no treatment, (**C**) 50 µg/g, (**D**) 100 µg/g, (**E**) 200 µg/g, and (**F**) 400 µg/g FV for 2 days. For oxidative stress induction, L4 nematodes were incubated on NGM agar containing 20 mM of PQ for 24 h. Unstressed control worms were incubated on NGM for 24 h. Representative images of fluorescent protein expression were taken. (**G**) Mean FI of GST-4::GFP was quantified and normalized to the control. Differences from control and PQ treatment were analyzed using one-way ANOVA with Tukey’s multiple comparison test. Data are presented as box plots showing the median, interquartile range (IQR), whiskers (min/max values), and individual data points from three independent experiments with 20 individual worms per condition. Significant *p* values are indicated as ** (*p* ≤ 0.01), **** (*p* ≤ 0.0001), or ns (not significant).

Given that the beneficial effects of AN and FV under PQ-induced oxidative stress were not fully explained by changes at the transcriptional level, we sought to evaluate their influence on the protein expression of GST-4, a key oxidative stress response gene. GST-4 is a detoxification enzyme that conjugates glutathione to ROS and serves as a well-established marker of oxidative stress resistance in *C. elegans* [31]. Therefore, we examined the transgenic *C. elegans* strain carrying the GFP-reporter gene gst-4 after oxidative stress induction with 20 mM PQ. Representative images of adult transgenic nematodes pre-treated with 50, 100, 200, and 400 µg/g AN or FV for 48 h, followed by a 24 h PQ exposure, are shown in Figure 5 and Figure 6.

Under unstressed conditions, AN and FV treatment did not significantly alter GST-4::GFP expression compared to untreated controls. Exposure to PQ strongly increased GST-4::GFP fluorescence intensity (FI) 4.8-fold (Figure 5) and 5.5-fold (Figure 6), indicating an activation of stress response pathways. Notably, pre-treatment with 400 µg/g of both extracts significantly attenuated the PQ-induced upregulation of GST-4::GFP. Specifically, AN reduced the mean FI to a 3.7-fold increase (*p* ≤ 0.01; Figure 5), while FV reduced it to 4.6-fold (*p* ≤ 0.01; Figure 6).

These findings suggest that AN and FV may enhance stress resilience in *C. elegans* by mitigating excessive stress signaling at the protein level, rather than primarily through transcriptional regulation.

## 3. Discussion

*Ascophyllum nodosum and Fucus vesiculosus* have great potential for use as feed supplements due to their health-promoting properties, including antioxidant, anti-inflammatory, antimicrobial, and antidiabetic properties [11]. However, precise mechanisms underlying the protective effects of brown algae are rarely described in the literature. In this study, we investigated the antioxidant potential of aqueous AN and FV extracts using both in vitro, and in vivo approaches to gain mechanistic insights into their mode of action.

Oxidative stress, characterized by excessive accumulation of ROS, plays a central role in promoting DNA damage, inflammation, and various chronic diseases [13]. Plant-derived bioactive compounds, including marine sources, have been shown to counteract oxidative damage [32,33]. Here, we demonstrate that both AN and FV extracts significantly reduced ROS levels in a concentration-dependent manner in Caco-2 and IPEC-J2 cells under oxidative stress, with FV exhibiting a more pronounced effect than AN at equivalent concentrations. These findings provide evidence of intracellular antioxidant activity for both species in intestinal epithelial models, complementing the existing literature, which has largely focused on chemical assays or different cell lines [12,34,35].

Previous research assessed the antioxidant effects of *Ascophyllum nodosum* and *Fucus vesiculosus* in Caco-2 cells using accelerated solvent extraction (ASE^®^). Antioxidant status was evaluated by measuring catalase (CAT), superoxide dismutase (SOD), glutathione (GSH) levels, and DNA protection via comet assay. Results indicated that *Ascophyllum nodosum* offered significant protection against H_2_O_2_-induced DNA damage, supporting their intracellular antioxidant potential. Similarly, *Fucus vesiculosus* has shown antioxidant activity in RAW 264.7 macrophages by reducing oxidative damage [36], though these studies did not involve intestinal cells or quantification of intracellular ROS accumulation.

The greater antioxidant capacity of FV correlates with its higher TPC, TEAC, and FRAP, which were approximately threefold higher than those of AN. As PTs are the predominant phenolic compounds in brown algae [12], we further characterized their composition using LC-HRMS/MS. This analysis confirmed the presence of PTs in both extracts, with FV exhibiting greater structural diversity, comprising 33 distinct PTs with degrees of polymerization (DP) ranging from 2 to 9. In contrast, only 10 PTs were detected in AN, with DPs ranging from 2 to 7. Although complete structural elucidation was not possible due to the complexity of the mixtures, the absence of separation techniques for individual components, and the lack of commercially available phlorotannin standards [12], the number and range of detected compounds provide valuable insights into the compositional differences between the extracts.

These results align with previously reported findings. For example, UHPLC-DAD-ESI-MSn analysis of the ethyl acetate fraction from a hydroacetonic extract of *Fucus vesiculosus* identified various PT subclasses, including fucols, fucophlorethols, and fuhalols, with DPs extending up to 22. Moreover, novel structures such as fucofurodiphlorethol and fucofuropentaphlorethol were tentatively identified [37]. Although the aqueous extract analyzed in this study showed a narrower DP range, it still demonstrated substantial chemical diversity, indicating that lower-molecular-weight PTs are sufficiently water-soluble to be recovered by aqueous extraction. Likewise, natural deep eutectic solvents (NADESs) combined with ultrasonic-assisted extraction (UAE) have been used to isolate 32 PTs from *Fucus vesiculosus*, identifying trimers to nonamers via HPLC-HRMS/MS [38], supporting the robustness of our aqueous extraction method in recovering functionally relevant compounds.

In contrast, PTs isolated from *Ascophyllum nodosum* using ethanol extraction followed by Sephadex LH-20 fractionation and LC-MS^n^ have been reported to include higher-molecular-weight oligomers with DPs ranging from 10 to 31 with ether bonds, dibenzodioxin structures, and sulfated derivatives [39]. Although our milder, water-based extraction method did not yield such high-DP PTs, it effectively extracted low- to mid-molecular-weight PTs, supporting the use of aqueous extraction for obtaining bioactive constituents, especially in the context of nutraceutical or feed applications.

Nonetheless, it is important to recognize that aqueous extraction is not selective for phenolics. Water also solubilizes a range of other bioactive molecules, notably, polysaccharides such as fucoidans, laminarins, and alginates. These polysaccharides are known for their diverse biological activities, including antioxidant, antitumor, antibacterial, antiviral, and anti-inflammatory properties [40,41,42]. Our chemical analyses confirmed that AN had a significantly higher total polysaccharide content (71 mg GE/g) compared to FV (48 mg GE/g), suggesting that polysaccharides may contribute substantially to the antioxidant effects observed in AN.

While in vitro assays provide useful preliminary data, they often fail to capture the complexity of biological systems and the bioavailability or metabolism of antioxidant compounds [43]. Therefore, we evaluated the effects of AN and FV in the model organism *C. elegans* to gain insights into their antioxidant activity in a whole-organism context. Despite the growing number of studies investigating brown algae extracts in vitro, evidence from *C. elegans* models remains limited.

We performed an oxidative stress assay using PQ, a redox-cycling herbicide that induces ROS production primarily via superoxide anion (O_2_•^−^) and hydrogen peroxide (H_2_O_2_) generation [44,45]. Worm motility, a sensitive indicator of neuromuscular health and overall vitality [46], was measured under both oxidative stress and unstressed conditions to assess extract efficacy. Under PQ-induced oxidative stress, both extracts conferred protective effects, but with distinct profiles. FV revealed modest effects of improved motility at 200 and 400 µg/g (12% and 11%, respectively, compared to PQ control). AN significantly preserved motility at the highest concentration (400 µg/g), resulting in a 22% improvement compared to the PQ control. These results suggest that AN may contain more potent or bioavailable constituents capable of mitigating acute oxidative damage in *C. elegans*, potentially through ROS scavenging or activation of endogenous stress response pathways.

Notably, these findings contrast with the in vitro ROS assays in intestinal epithelial cells, where FV exhibited greater antioxidant efficacy. One plausible explanation is that FV’s structurally diverse PTs, while potent antioxidants in vitro, may have limited bioavailability or be metabolized less efficiently in *C. elegans*. Conversely, AN’s bioactivity may be driven not only by its PTs but also by its relatively higher polysaccharide content, such as fucoidans and alginates, which are known to exhibit antioxidant, immunomodulatory, and stress-protective effects. These polysaccharides may enhance bioavailability or stability or synergize with phenolics to more effectively mitigate oxidative damage in the worm.

This discrepancy between AN and FV effects across cell-based and *C. elegans* model underscores the importance of in vivo studies to fully elucidate the biological activity and bioavailability of seaweed-derived compounds, as different model systems may engage distinct oxidative stress pathways or metabolize extract constituents differently.

Moreover, such differences also reflect the complexity of organismal physiology versus isolated cellular models. While in vitro systems provide a simplified environment to assess direct antioxidant effects, whole-organism models such as *C. elegans* integrate systemic factors including digestion, metabolism, and tissue-specific responses, which may influence the observed efficacy of bioactive compounds. Thus, cellular antioxidant capacity may not always correlate with organismal resilience.

Under non-stressed conditions, FV exhibited greater efficacy in supporting long-term motility, with significant improvements observed at both 100 µg/g and 400 µg/g. AN also enhanced motility, but only at the highest dose. These findings indicate that while AN is more effective during acute oxidative challenge, FV may promote healthspan by supporting physiological function in the absence of external stressors.

Our findings are consistent with previous research highlighting the antioxidant potential of AN in *C. elegans*, particularly under conditions of physiological stress. For instance, a fucose-containing polymer (FCP) derived from AN significantly enhanced the survival of *C. elegans* under thermal stress, with survival increases of 24–27% depending on the severity of heat exposure [29]. Similarly, treatment with the commercial AN extract Tasco^®^ was shown to extend mean and maximum lifespan by approximately 17% and to improve neuromuscular function, as measured by the pharyngeal pumping rate [28]. These findings confirm the perception that AN exerts beneficial effects not only in lifespan extension but also in maintaining organismal vitality under stress, mirroring our results in the PQ-induced oxidative stress model.

In contrast, fewer studies have investigated the effects of FV in *C. elegans*. However, our observation that FV supports motility under unstressed conditions aligns with its documented neuroprotective and mitochondrial-supportive properties in other model systems. For example, FV-derived fucoidan has been shown to alleviate mitochondrial dysfunction and prevent dopaminergic neuron loss in a Parkinson’s disease mouse model, effects mediated via the regulation of ATP5F1a, a key mitochondrial protein [47].

**Figure 7:**
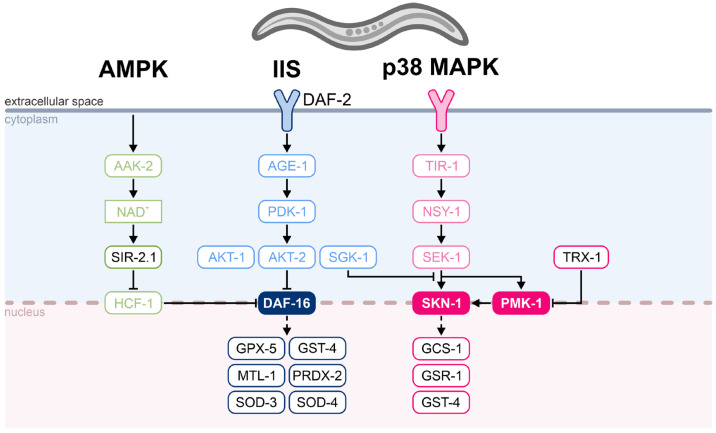
Overview of oxidative stress response signaling pathways and downstream targets in *C. elegans*. The AMPK (green), insulin/IGF-1 signaling (IIS, blue), and p38 MAPK (pink) pathways regulate oxidative stress responses through transcription factors DAF-16 and SKN-1. Activation of AMPK via AAK-2 and SIR-2.1 promotes DAF-16 translocation and activity. The IIS pathway, mediated by DAF-2 and downstream kinases (AGE-1, PDK-1, AKT-1/2, SGK-1), inhibits DAF-16 under favorable conditions. In parallel, the p38 MAPK cascade, involving TIR-1, NSY-1, and SEK-1, activates SKN-1 and PMK-1 to induce detoxification and antioxidant genes. DAF-16 targets include *gpx-5*, *mtl-1*, *sod-3*, *sod-4*, *prdx-2*, and *gst-4*, while SKN-1 and PMK-1 regulate *gcs-1*, *gsr-1*, and *gst-4*. *trx-1*, part of the thioredoxin system, modulates redox homeostasis and may interact with p38 MAPK components. Tested target genes are shown in black font, and transcription factors are shown with filled boxes.

To investigate whether brown algae extracts modulate key molecular pathways involved in oxidative stress responses, we analyzed the expression of 14 representative stress-related genes under both unstressed and oxidative stress conditions using RT-qPCR. In *C. elegans*, oxidative stress activates conserved defense mechanisms that maintain cellular homeostasis and promote survival [44]. These responses are coordinated primarily by three signaling pathways: AMP-activated protein kinase (AMPK), insulin/IGF-1 signaling (IIS), and the p38 mitogen-activated protein kinase (p38 MAPK) cascade. These pathways converge on the transcription factors DAF-16 and SKN-1, which control a wide array of stress-responsive genes involved in redox balance, detoxification, metal ion homeostasis, and repair [48]. DAF-16, modulated by the AMPK effector *sir-2.1* and the IIS receptor *daf-2*, induces key antioxidant and cytoprotective genes including *gpx-5* (glutathione peroxidase), *mtl-1* (metallothionein), *sod-3* and *sod-4* (superoxide dismutases), *prdx-2* (peroxiredoxin), and *gst-4* (glutathione S-transferase) [49]. These enzymes contribute to the neutralization of ROS such as superoxide (O_2_•^−^) and hydrogen peroxide (H_2_O_2_) and aid in the removal of toxic byproducts [50]. In parallel, the p38 MAPK pathway activates SKN-1 and PMK-1, which upregulate genes such as *gcs-1* (involved in glutathione biosynthesis), *gsr-1* (glutathione reductase), and *gst-4*, further supporting detoxification and oxidative defense [51,52]. The gene *trx-1*, a component of the thioredoxin system, contributes to protein redox regulation and may modulate stress signaling via cross-talk with MAPK pathways [53]. The selected target genes and their respective pathways are highlighted in Figure 7.

Our results indicate that both AN and FV moderately activate components of the antioxidant defense network under unstressed conditions. Treatment with AN at 100 µg/g significantly increased the expression of *gsr-1* and *trx-1*, suggesting enhanced glutathione regeneration and redox cycling. At 400 µg/g, AN maintained *gsr-1* upregulation and showed a slight downregulation of *sir-2.1*, possibly reflecting subtle modulation of the IIS or AMPK signaling. FV treatment elicited a broader transcriptional response, particularly at 400 µg/g, where significant upregulation of *daf-16*, *gpx-5*, *pmk-1*, *sod-3*, and *sod-4* was observed. These gene expression changes point to activation of the IIS and p38 MAPK pathways and suggest that FV may enhance both mitochondrial and extracellular ROS detoxification.

However, under PQ-induced oxidative stress, neither AN nor FV significantly altered the robust transcriptional response triggered by PQ exposure. This suggests that the protective effects observed were not mediated through additional transcriptional upregulation of the tested antioxidant genes. However, it remains possible that transcriptional changes occur within a narrower temporal window that was not captured in our 24 h post-treatment analysis. For instance, early transcriptional modulation at 6 h may be transient and no longer detectable at the later time point. Therefore, the involvement of transcriptional mechanisms cannot be fully excluded. Alternatively, the observed protective effects may primarily result from non-transcriptional mechanisms such as direct radical scavenging or modulation of stress-signaling proteins.

To investigate this further, we assessed stress response at the protein level using a transgenic *C. elegans* strain expressing *gst-4*::GFP. Pre-treatment with 400 µg/g of both extracts significantly attenuated the PQ-induced upregulation of GST-4::GFP. These findings suggest that AN and FV may enhance stress resilience in *C. elegans* by mitigating excessive stress signaling at the protein level, rather than primarily through transcriptional regulation.

Notably, our study is among the first to directly compare the antioxidant activities of AN and FV in *C. elegans* using worm motility as a functional endpoint under both stress and non-stress conditions. While previous studies have separately examined the longevity-enhancing or stress-mitigating effects of AN and the neuroprotective actions of FV in other models, none have systematically evaluated these extracts side by side in *C. elegans* using an oxidative stress assay such as PQ-induced ROS generation.

In summary, our study provides mechanistic insights into the antioxidant potential of *Ascophyllum nodosum* and *Fucus vesiculosus* by combining in vitro cellular assays, targeted phlorotannin profiling, and in vivo evaluation in *C. elegans*. While FV showed greater antioxidant activity in intestinal cell models, AN was more effective in preserving motility under oxidative stress in *C. elegans*. Both extracts attenuated stress-induced protein-level responses without significantly altering antioxidant gene expression under stress, suggesting non-transcriptional mechanisms of action. The differential responses observed across model systems underscore the complexity of bioavailability and metabolic fate of algal compounds. Future studies should focus on identifying individual bioactive components and clarifying their in vivo mechanisms of action to support the development of seaweed-derived functional ingredients for animal health.

## 4. Materials and Methods

### 4.1. Brown Algae Extract Preparation

Dried AN powder (particle size 100 µm) and FV meal (particle size 1–2 mm) were provided by NUQO^©^. The AN sample was supplied as a pre-micronized powder, eliminating the need for further particle size reduction. In contrast, FV meal was ground using a coffee mill to achieve a comparable particle size prior to extraction. Solid–liquid extraction was performed to obtain extracts from both AN and FV. Brown alga powder was mixed with dH_2_O at a ratio of 1:10 *w*/*w*. Deionized water was selected as the extraction solvent due to its non-toxic, food-safe properties and relevance for applications in animal feed, where organic solvent residues would be undesirable. Extractions were conducted at room temperature under continuous overhead agitation for 24 h. Following extraction, the mixtures were centrifuged at 16,000× *g* for 30 min, and the supernatants were collected. The dry mass of the extracts was measured using an IR Moisture Analyzer (Sartorius, Göttingen, Germany). Liquid extracts were stored at −20 °C until further analysis. In this study, the reported concentrations of brown algae extracts correspond to the initial macroalgal material in water.

### 4.2. TPC of Brown Algae Extracts

The TPC of the AN and FV extracts was determined using the Folin–Ciocalteu method previously described [54]. The phlorotannin monomer phloroglucinol served as the external standard for quantifying phenolic compounds in the brown algae extracts. To create the calibration curve, a phloroglucinol stock solution (1 g/L, Sigma-Aldrich, St. Louis, MO, USA) was serially diluted with dH_2_O (200, 150, 100, 80, 60, 40, 20, 10 mg/L). For the analysis, 1 mL of dH_2_O, 100 μL of the AN or FV extract (diluted 100-fold) or standard solution, 100 μL of Folin–Ciocalteu reagent (Sigma-Aldrich), and 500 μL of saturated sodium carbonate solution (Sigma-Aldrich) were combined in microcentrifuge tubes. The mixtures were vortexed immediately and then incubated in the dark at room temperature for 70 min. After incubation, 200 µL of the sample was transferred to the wells of a clear 96-well microplate (Greiner Bio-One GmbH, Kremsmünster, Austria), and the absorbance was measured at 750 nm using a POLARstar Omega microplate reader system (BMG Labtech, Ortenberg, Germany). All measurements were carried out in well triplicates. The phloroglucinol calibration curve was used to calculate the TPC for AN and FV extracts, which is expressed as mg of phloroglucinol equivalents (PGE) per g of sample (mg PGE/g).

### 4.3. TPSC of Brown Algae Extracts

The TPSC of the AN and FV extracts was determined using the phenol-sulfuric acid method previously described [55]. For the standard curve, a D-glucose solution (1 g/L, Sigma-Aldrich) was serially diluted with dH_2_O (150, 100, 80, 60, 40, 20, 10 mg/L). For the analysis, 200 µL of dH_2_O, AN or FV extract (diluted 100-fold) or standard solution, 600 μL of concentrated sulfuric acid (Sigma-Aldrich), and 120 μL of 5% phenol solution (Sigma-Aldrich) were mixed in reaction tubes with screw caps. The mixtures were vortexed immediately and then incubated in a thermoshaker at 85 °C for 20 min. After incubation, 230 µL of the samples were transferred to the wells of a clear 96-well microplate, and the absorbance was measured at 490 nm using a POLARstar Omega microplate reader system. All measurements were carried out in well triplicates. The glucose calibration curve was used to calculate the TPSC for AN and FV extracts, which is expressed as mg of glucose equivalents (GE) per g of sample (mg GE/g).

### 4.4. TEAC of Brown Agae Extracts

The TEAC of the AN and FV was determined as described previously [56,57] with modifications [58]. Trolox was used as an antioxidant standard. For the standard curve, a Trolox stock solution (1 mol/L, Sigma-Aldrich) was serially diluted with water (750, 500, 250, 100, 50, 25 mmol/L). ABTS, 2,2′-azinobis(3-ethylbenzothiazoline-6-sulfonic acid) diammonium salt (Sigma-Aldrich) was dissolved in water to a concentration of 7 mM. ABTS radical cation (ABTS^•+^) was produced by adding potassium persulfate (Sigma-Aldrich) to the ABTS stock solution to a final concentration of 2.45 mM. The mixture was incubated in the dark at room temperature for 16 h before use. For analysis, ABTS containing K_2_S_2_O_8_ solution was diluted with dH_2_O to an absorbance of 0.70 (±0.02) measured at 734 nm. Next, 196 µL of this ABTS solution were mixed with 4 µL standard, AN or FV extract diluted by a factor of 10, or dH_2_O (blank) in the wells of a clear 96-well microplate, and incubated for 5 min at room temperature, and the absorbance was measured at 734 nm using a POLARstar Omega microplate reader system. All measurements were carried out in well triplicates. The Trolox calibration curve was used to calculate the antioxidant activity for AN and FV, which is expressed as µM of Trolox equivalents (TE) per g of sample (µmol TE/g).

### 4.5. FRAP of Brown Algae Extracts

The FRAP of AN and FV was assayed as described with slight modifications [59]. Trolox was used as an antioxidant standard. For the standard curve, a Trolox stock solution (1 mol/L) was serially diluted with water (750, 500, 250, 100, 50, 25 mmol/L). The oxidant used in the FRAP assay was a reagent mixture prepared by combining 300 mM acetate buffer (pH 3.6), 20 mM ferric chloride solution, and 10 mM TPTZ solution (dissolved in 40 mM HCl, Sigma-Aldrich) in a 10:1:1 ratio, respectively, before application. The FRAP reagent was heated to 37 °C while protected from light. For analysis, 20 μL of dH_2_O (blank), standard, or diluted sample and 180 μL of FRAP reagent were mixed in a 96-well microplate. The absorbances were measured at 593 nm after 10 min using a POLARstar Omega microplate reader system. All measurements were carried out in well triplicates. The Trolox standard curve was used to calculate the antioxidant activity of the samples and was expressed as µM Trolox equivalents (TE) per g of sample (μmol TE/g).

### 4.6. LC-HRMS/MS Analysis

Phlorotannin profiles of AN and FV extracts were characterized using targeted and untargeted LC-HRMS/MS analysis to identify and annotate potential phlorotannin compounds. Freeze-dried AN and FV extracts were solved in acetonitrile–water (10:90, *v*/*v*) to a final concentration of 5 mg/mL and centrifuged, and the supernatant was transferred into glass LCMS vials. Each sample was prepared in triplicate, and a volume of 1 μL was injected for LC-HRMS/MS analysis. In detail, the high-performance liquid chromatograph Agilent 1260 Infinity (Agilent Technologies, Waldbronn, Germany), consisting of an on-line degasser, a binary pump, a high-performance SL autosampler, and a thermostated column compartment, was coupled to the LTQ VelosPro Orbitrap Elite mass spectrometer with an ESI source (Thermo Fisher Scientific, San Jose, CA, USA). For chromatographic separation, the Ascentis Express RP-Amide 10 cm × 2.1 mm column with a 2 μm particle size (Sigma-Aldrich) was utilized and kept at 30 °C during gradient elution with a flow rate of 0.25 mL/min. The eluent constitution was acetonitrile with 0.2% formic acid (*v*/*v*, eluent B) and water with 0.2% formic acid (*v*/*v*, eluent A). The gradient started with 10% B at 0 min, followed by an increase in eluent B to 100% at 36 min and holding B at 100% for 14 min; the column was equilibrated from 50 to 60 min at 10% of eluent B. The mass spectra were recorded in data-dependent mode, acquiring profile full-scan spectra with a resolving power setting of 30,000 FWHM (at 400 *m/z*) and a scan range of *m/z* 120–1200 in the negative mode. The fragment MS/MS spectra of the four most intense ions after higher-energy collisional dissociation were collected using the following settings: normalized collision energy of 55 eV, isolation width 2 Da, minimum signal of 50,000, and resolving power setting of 15,000 FWHM (at 400 *m/z*). A rejection mass list was created from the most intense ions observed in blank sample, which were found in averaged mass spectra across the whole chromatogram and applied. The ESI source parameters were set as follows: capillary voltage 3.5 kV, capillary temperature 350 °C, sheath gas 50, aux gas 12, and sweep gas 1 arbitrary units.

The thermo.raw files were processed in Compound Discoverer software v3.3.1.111 (Thermo Fisher Scientific). First, a list of possible PTs was created based on the common conjugation described in the literature [60] using the transformation reactions within Compound Discoverer software. These expected compounds, in an in silico-generated mass list, containing 192 unique molecular formulas and exact masses calculated in the range of 2–20 phloroglucinol units (C_12_H_10_O_6_-C_120_H_78_O_60_), were used for targeted detection. Moreover, typical neutral losses of C_6_H_4_O_3_, C_6_H_5_O_3_, C_6_H_6_O_3_, C_6_H_8_O_4_, C_6_H_8_O_5_, and C_6_H_6_O_4_ associated with PTs were defined and used for the purposes of screening MS^2^ data. A modified Compound Discoverer workflow, combining detection of unknown features with blank background removal, expected compound search, and neutral loss search, was used. In more detail, features were detected within a mass accuracy tolerance of 5 × 10^−6^ (relative mass error) with a minimal peak intensity of 3000 and chromatographic S/N threshold of 3. One of the following adducts was assigned: (2M-H)^−^; (M+Cl)^−^; (M+HCOOH-H)^−^; (M-2H)^2−^; (M-H)^−^. The most intense feature was reported, and compounds were grouped across all samples using a mass tolerance of 5 × 10^−6^ (relative) and a retention time window of 0.1 min with preferred ions (M-2H)^2−^; (M-H)^−^. The detected compounds were searched against a pre-created mass list of PTs. All PTs were tagged based on the match with the mass list or presence of the typical neutral loss. The dataset was exported in an Excel file for further analysis, while the potential PTs were tagged.

### 4.7. Cell Culture Maintenance

Porcine IPEC-J2 and human Caco-2 epithelial cells were purchased from DSMZ (Braunschweig, Germany) and maintained in Dulbecco’s Modified Eagle Medium (DMEM, +4.5 g/L glucose, +stable glutamine, +sodium pyruvate. +3.7 g/L sodium bicarbonate), and Eagle Minimum Essential Medium (EMEM, +Earle’s balanced salts, +2 mM L-glutamine, +non-essential amino acid, +1 mM sodium pyruvate, +1.5 g/L sodium bicarbonate), respectively, supplemented with 10% [*v*/*v*] fetal bovine serum (FBS and 100 U/mL–100 μg/mL penicillin-streptomycin (P-S) (all from PAN-Biotech, Aidenbach, Germany). Cells were cultivated under standard conditions at 37 °C in a humidified atmosphere (≥95%) with 5% CO_2_ and were subcultured two times a week.

### 4.8. Cytotoxicity Assay

Cytotoxic effects of AN and FV were determined using a resazurin-based in vitro toxicology assay (Sigma-Aldrich) according to the manufacturer’s instructions. Briefly, cells were seeded in triplicate into clear 96-well plates at a density of 5 × 10^4^ (Caco-2) or 2 × 10^4^ (IPEC-J2) cells per well and grown overnight. The next day, the cells were washed with DPBS and incubated with medium containing 50, 100, 200, and 400 µg/g AN or FV for 24 h under standard conditions. After treatment, cells were washed with DPBS and incubated with medium containing 10% resazurin for 1.5 h. Fluorescence measurements of resorufin, the reduced form of resazurin, were obtained using a POLARstar Omega microplate reader with excitation at 544 nm and emission at 590 nm. Cell viability results were normalized against those of untreated control cells.

### 4.9. Quantification of Intracellular ROS Production in Caco-2 and IPEC-J2 Cells

Intracellular antioxidant activity was evaluated by measuring ROS levels with the cell-permeable probe H_2_DCF-DA, after inducing oxidative stress using the free radical generator AAPH in Caco-2 and IPEC-J2 cell lines. Intracellular H_2_DCF-DA is enzymatically cleaved and oxidized to the highly fluorescent 2′,7′-dichlorofluorescein (DCF) in the presence of ROS. The increase in FI is directly proportional to the amount of ROS generated, allowing for the quantification of intracellular ROS levels [61]. The procedure was performed as previously described [62].

In short, cells were seeded in triplicate into black 96-well plates (Greiner Bio-One GmbH) at a density of 5 × 10^4^ (Caco-2) or 2 × 10^4^ (IPEC-J2) cells per well and grown overnight. The following day, cells were washed with DPBS and co-treated with 100 µL of 50 µM H_2_DCF-DA (Sigma-Aldrich) and AN or FV (100, 200, and 400 µg/g in HBSS) at 37 °C for 20 min. QUE (10 µM, Sigma-Aldrich) was used as an antioxidant positive control. After treatment, cells were washed with DPBS and then stressed with 100 µL of 200 µM AAPH (Sigma-Aldrich) in HBSS. Cells treated with HBSS served as controls.

The amount of produced DCF was measured using a POLARstar Omega microplate reader in fluorescence mode with excitation at 485 nm and emission at 530 nm. Data were collected immediately following the addition of the stressor and subsequently at 15, 30, 60, and 90 min. The oxidative status over time was summarized by calculation of the AUC and normalization to the AAPH stress control.

### 4.10. C. elegans Strains and Maintenance

Nematode strains (wild-type Bristol N2, VP596 [*gst-4*::GFP], and SS104 [*glp-4*(bn2)]) were obtained from the *C. elegans* Genetics Center (CGC, University of Minnesota, MN, USA). The worms were cultivated as described [63] and maintained on 10 cm Nematode Growth Medium (NGM) agar plates seeded with Escherichia coli OP50 (CGC) at 20 °C, except for SS104, which was cultivated at 15 °C. All chemicals and reagents for cultivation of *C. elegans* were purchased from Carl Roth (Karlsruhe, Germany), if not stated otherwise.

### 4.11. C. elegans Synchronization

Nematodes were age-synchronized by washing gravid adult worms off NGM plates with S-Basal buffer containing 5 mg/mL PEG 3350 (Sigma Aldrich). The suspension was collected in a 15 mL reaction tube, and the worms were settled by gravitation followed by another washing step with S-Basal + PEG buffer to remove residual bacteria. Next, the nematodes were bleached with 1% [*v*/*v*] alkaline hypochlorite solution (1 mL 5% NaClO, 0.5 mL 5 M NaOH, 3.5 mL dH_2_O), and the eggs were released after approximately 5 min of constant vortexing at room temperature. After centrifugation at 1300× *g* for 1 min, the eggs were washed twice with 5 mL S-Basal + PEG buffer to remove residual bleaching solution.

### 4.12. Worm Toxicity Test

The potential toxic effects of AN and FV on *C. elegans* were evaluated by quantifying survival following exposure to each extract. Approximately 40 age-synchronized wild-type nematodes were transferred to 3 cm NGM agar plates seeded with OP50 and supplemented with AN or FV at concentrations of 100, 400, 800, 1600, and 10,000 µg/g. Worms were incubated at 20 °C for 72 h. After the exposure period, the numbers of live and dead worms were determined by visual inspection under a SZX16 stereomicroscope (Olympus, Tokyo, Japan). Each treatment was conducted in triplicate, and survival rates were calculated as the percentage of live worms relative to the total number assessed.

### 4.13. Monitoring Lifespan Worm Motily Under Unstressed Conditions

The effect of AN and FV treatment on nematode locomotion under unstressed conditions was determined via activity assay using the mutant *C. elegans* strain SS104. The temperature-sensitive defect in germ-line proliferation during larval development allowed for sterile nematode populations at 25 °C. Nematodes were age-synchronized, and eggs were transferred to an Erlenmeyer flask containing 30 mL liquid S medium prepared as described [63]. Eggs were incubated at 25 °C and left to hatch overnight. After 20 h, nematodes were fed with OP50 in a final concentration of 2.5 mg/mL in S medium. Nematodes were incubated for another 52 h at 25 °C. Next, at least 60 nematodes were transferred into the wells of a 12-well microplate containing AN, or FV (10, 100, 400 µg/g) in S medium seeded with 2.5 mg/mL OP50. No treatment was added to the wells containing the control worms. After 72 h incubation time at 25 °C, visual inspection of the nematodes verified that no offspring has been produced. Single nematodes were pipetted into each well of a black-bottom, low-volume 384-well microplate (Greiner Bio-One GmbH). In total, 20 nematodes per group were transferred into wells that had been filled up with 20 µL medium containing the respective concentration of AN and FV and 2.5 mg/mL OP50. The 384-well plate was covered with a plate lid, sealed with parafilm, and incubated at 25 °C. The motility of single nematodes in individual wells was monitored in regular time intervals for up to 28 days using WMicrotracker ONE (Phylumtech, Santa Fe, Argentina). The worm motility in each well was summarized by calculation of the AUC and was normalized to the control. Experiments were performed with 20 nematodes per treatment group.

### 4.14. Monitoring Worm Motility Under PQ-Induced Oxidative Stress

The alteration of nematode locomotion under stressed conditions with or without AN and FV treatment was evaluated via activity assay. Age-synchronized wild-type N2 nematodes were incubated on 6 cm NGM plates seeded with OP50 and supplemented with 100, 200, or 400 µg/g of AN and FV at 20 °C for 3 days until adulthood. Adult worms were then washed off plates using S-Basal medium supplemented with PEG (0.5% [*w*/*v*]) and 5 mg/mL cholesterol in ethanol. Worms (80 per well) were transferred into clear 96-well microplates in 95 µL S-Basal + PEG + cholesterol buffer. The basal activity of nematodes in each well was monitored for 1 h using WMicrotracker ONE and used for normalization to account for differences in worm numbers between wells. Oxidative stress was induced by adding 5 µL of PQ (1M stock in dH_2_O, Sigma-Aldrich) to each well, resulting in a final concentration of 50 mM. Control wells received 5 µL of dH_2_O instead. Worm motility in each well was recorded every 5 min for a total of 48 h and summarized by calculating the AUC, normalized to the control. Experiments were performed with eight wells per treatment group.

### 4.15. Gene Expression Analysis

AN and FV were examined for their regulatory effects on oxidative stress-related gene expression via quantitative real-time PCR. Age-synchronized wild-type N2 nematodes were incubated on 6 cm NGM plates seeded with OP50 and supplemented with either 100 µg/g or 400 µg/g of AN or FV at 20 °C for 48 h, until they reached the L4 stage. Approximately 1000 nematodes were then transferred to fresh OP50-seeded 6 cm NGM plates containing either 20 mM PQ for oxidative stress induction or control plates without PQ for unstressed conditions. The worms were further incubated at 20 °C for 24 h until they reached adulthood.

For RNA isolation, adult nematodes were washed off the plates using S-Basal + PEG buffer and enzymatically lysed using a modified protocol with Proteinase K (New England BioLabs, Ipswich, MA, USA) [64]. Total RNA was isolated using the RNeasy^®^ Plus Kit (Qiagen, Hilden, Germany) according to the manufacturer’s instructions. RNA concentration and the 260/280 nm ratio were measured using a POLARstar Omega microplate reader.

For gene expression analysis, 200 ng of total RNA was transcribed into cDNA using the iScript cDNA Synthesis Kit (Bio-Rad Laboratories, Hercules, CA, USA) according to the manufacturer’s instructions. cDNA synthesis was performed in a CFX Connect or CFX96 Real-Time System (Bio-Rad Laboratories) thermal cycler with the following protocol: priming at 25 °C for 5 min, reverse transcription at 46 °C for 20 min, and reverse transcriptase inactivation at 95 °C for 1 min. The resulting cDNA was diluted with DEPC-H_2_O to a final concentration of 2.5 ng/μL for subsequent qPCR analysis.

mRNA expression levels of genes involved in oxidative stress were quantitatively measured by real-time PCR using iQ SYBR Green Supermix (Bio-Rad Laboratories). qPCR was performed using a thermal cycler with the following protocol: DNA denaturation and polymerase activation at 95 °C for 3 min, followed by 40 amplification cycles of denaturation at 95 °C for 10 s, primer annealing at 57.5 °C for 30 s, and extension at 72 °C for 20 s. Melt curve analysis was conducted by gradually increasing the temperature from 65 °C to 95 °C in 0.5 °C increments.

Each experiment was performed in triplicate plates per day, with samples measured in technical duplicates. Gene expression data were normalized to multiple reference genes: beta Actin (*act-1*), DNA-directed RNA polymerase II subunit RPB1 (*ama-1*), and Peroxisomal Membrane Protein-related protein (*pmp-3*). Target genes for *C. elegans* included insulin-like receptor (*daf-2*), fork head-related transcription factor (*daf-16*), glutamate-cysteine ligase (*gcs-1*), glutathione peroxidase 5 (*gpx-5*), glutathione disulfide reductase 1 (*gsr-1*), glutathione S-transferase 4 (*gst-4*), metallothionein-1 (*mtl-1*), mitogen-activated protein kinase pmk-1 (*pmk-1*), peroxiredoxin prdx-2 (*prdx-2*), yeast SIR related 2.1 (*sir-2.1*), protein skinhead-1 (*skn-1*), mitochondrial superoxide dismutase [Mn] 2 (*sod-3*), extracellular superoxide dismutase [Cu-Zn] (*sod-4*), and thioredoxin 1 (*trx-1*). Relative mRNA expression levels of the target genes were calculated using the 2^−ΔΔcT^ method [65].

Primers for oxidative stress were designed and evaluated according to MIQE guidelines [66]. Genes used in the experiments were selected based on a literature search. Primers were ordered from Microsynth AG (Balgach, Switzerland), and their sequences are listed in Table 3.

### 4.16. Protein Expression of GFP-Labeled GST-4 in Transgenic Nematodes

The effect of AN and FV on oxidative stress responses was assessed via GFP fluorescence detection of the reporter gene *gst-4*. Age-synchronized, VP596 transgenic nematodes (*gst-4*::GFP) were incubated on 10 cm NGM plates seeded with OP50 and supplemented with 50, 100, 200, or 400 µg/g of AN or FV at 20 °C for 48 h until the nematodes reached the L4 stage. Subsequently, approximately 1000 nematodes were transferred to fresh 6 cm OP50-seeded NGM plates containing either 20 mM PQ for oxidative stress induction or control plates without PQ for unstressed conditions. The worms were further incubated at 20 °C for 24 h until adulthood. After incubation, nematodes were washed and anesthetized using a 1 mM levamisole hydrochloride solution (abcam, Cambridge, UK) for 5 min before microscopic imaging. GFP fluorescence of the *gst-4* reporter gene was visualized using an Olympus SZX16 stereomicroscope equipped with a pE-300 white illumination system (Cool LED, Andover, UK) and a GFP filter set. Images were captured using an Orca-R2 CCD camera (Hamamatsu Photonics, Shizuoka, Japan) with a 2× air objective. A minimum of 20 individual worms per treatment and experiment day were imaged. Mean FI of GST-4::GFP was quantified across the whole worm body using *C. elegans* analysis software [67]. Correlations between worm size and mean FI were assessed using Pearson correlation analysis to exclude biased data interpretation. While statistical evaluation was performed on raw images. Displayed images were background-corrected using ImageJ (version 1.53), and the green-fire-blue pseudo-color look-up table (LUT) was selected. Additionally, an overlay using white-light images with 15% transparency was generated.

### 4.17. Statistics

Statistical analysis was performed using Graphpad Prism version 9.3.1 (GraphPad Software, San Diego, CA, USA). Outliers were identified using the ROUT outliers test, and data was checked for normal distribution. ANOVA followed by Dunnett’s or Tukey’s multiple comparison test was used to compare more than two groups. Significant *p* values were indicated as * (*p* ≤ 0.05), ** (*p* ≤ 0.01), *** (*p* ≤ 0.001) or **** (*p* ≤ 0.0001). The graphical abstract was created with BioRender.com (Science Suite Inc., Toronto, ON, Canada). Figures were prepared in CorelDRAW^®^ Graphics Suite, Version 21.0.0.593 (Corel Corporation, Ottawa, ON, Canada).

## 5. Conclusions

This study demonstrates the antioxidant potential of *Ascophyllum nodosum* (AN) and *Fucus vesiculosus* (FV) through complementary in vitro and in vivo approaches, including ROS quantification in intestinal epithelial cells and motility assays in *C. elegans* under oxidative stress. FV exhibited stronger intracellular antioxidant activity in vitro, correlating with its higher phlorotannin content and structural diversity as identified by LC-HRMS/MS. In contrast, AN more effectively preserved *C. elegans* motility during oxidative challenge, indicating differences in bioavailability, metabolism, or underlying mechanisms of antioxidant action. Gene expression analyses revealed modest activation of antioxidant pathways under unstressed conditions, while stress-induced protein-level responses, measured via *gst-4::GFP* expression, were significantly attenuated by both extracts.

These findings underscore the importance of integrated cellular and organismal models in fully capturing the differential bioactivities of marine-derived compounds. Based on their demonstrated efficacy and composition, both AN and FV show strong potential as natural feed additives to support antioxidant defense and stress resilience in animal health. Future studies should focus on isolating active constituents and elucidating their bioavailability and mode of action in vivo.

## Figures and Tables

**Table 1:** Tentatively identified PTs in aqueous AN extract with chemical formula, calculated molecular weight (MW), detected *m*/*z*, retention time (RT), detected MS^2^ fragments, and reference ion.

	Formula	Calc. MW	*m/z*	RT [min]	DP	MS^2^	Reference Ion
PT 1	C_12_H_10_O_6_	250.05	249.04	2.95	2	No MS^2^	[M-H]^−^
PT 2	C_18_H_14_O_9_	374.06	373.06	6.16	3	No MS^2^	[M-H]^−^
PT 3	C_24_H_18_O_12_	498.08	497.07	3.70	4	No MS^2^	[M-H]^−^
PT 4	C_24_H_18_O_12_	498.08	497.07	9.42	4	No MS^2^	[M-H]^−^
PT 5	C_24_H_18_O_12_	498.08	497.07	3.49	4	No MS^2^	[M-H]^−^
PT 6	C_24_H_18_O_12_	498.08	497.07	1.81	4	No MS^2^	[M-H]^−^
PT 7	C_30_H_22_O_15_	622.09	621.09	7.13	5	No MS^2^	[M-H]^−^
PT 8	C_30_H_22_O_15_	622.10	621.09	2.99	5	No MS^2^	[M-H]^−^
PT 9	C_36_H_26_O_18_	746.11	745.10	10.27	6	No MS^2^	[M-H]^−^
PT 10	C_42_H_30_O_21_	870.13	869.12	11.41	7	DDA for preferred ion	[M-H]^−^

**Table 2:** Tentatively identified PTs in aqueous FV extract with chemical formula, calculated molecular weight (MW), detected *m/z*, retention time (RT), detected MS^2^ fragments, and reference ion.

	Formula	Calc. MW	*m/z*	RT [min]	DP	MS^2^	Reference Ion
PT 1	C_12_H_10_O_6_	250.05	249.04	1.42	2	No MS^2^	[M-H]^−^
PT 2	C_18_H_10_O_9_	370.03	369.02	13.45	3	No MS^2^	[M-H]^−^
PT 3	C_18_H_14_O_9_	374.06	373.06	1.41	3	DDA for preferred ion	[M-H]^−^
PT 4	C_18_H_14_O_9_	374.06	373.06	2.19	3	DDA for preferred ion	[M-H]^−^
PT 5	C_18_H_14_O_9_	374.06	373.06	6.15	3	No MS^2^	[M-H]^−^
PT 6	C_24_H_18_O_12_	498.08	497.07	9.26	4	No MS^2^	[M-H]^−^
PT 7	C_24_H_18_O_12_	498.08	497.07	2.01	4	DDA for preferred ion	[M-H]^−^
PT 8	C_24_H_18_O_12_	498.08	497.07	1.78	4	No MS^2^	[M-H]^−^
PT 9	C_24_H_18_O_12_	498.08	497.07	1.33	4	No MS^2^	[M-H]^−^
PT 10	C_60_H_42_O_30_	1242.17	620.08	10.73	5	No MS^2^	[M-2H]^2−^
PT 11	C_30_H_22_O_15_	622.09	621.09	2.96	5	DDA for preferred ion	[M-H]^−^
PT 12	C_30_H_22_O_15_	622.09	621.09	4.09	5	No MS^2^	[M-H]^−^
PT 13	C_30_H_22_O_15_	622.10	621.09	11.46	5	No MS^2^	[M-H]^−^
PT 14	C_30_H_22_O_15_	622.10	621.09	2.17	5	No MS^2^	[M-H]^−^
PT 15	C_30_H_22_O_15_	622.10	621.09	1.92	5	No MS^2^	[M-H]^−^
PT 16	C_36_H_26_O_18_	746.11	745.10	12.80	6	No MS^2^	[M-H]^−^
PT 17	C_36_H_26_O_18_	746.11	745.10	12.57	6	No MS^2^	[M-H]^−^
PT 18	C_36_H_26_O_18_	746.11	745.10	6.69	6	DDA for preferred ion	[M-H]^−^
PT 19	C_36_H_26_O_18_	746.11	745.10	3.19	6	No MS^2^	[M-H]^−^
PT 20	C_36_H_26_O_18_	746.11	745.10	5.19	6	No MS^2^	[M-H]^−^
PT 21	C_36_H_26_O_18_	746.11	745.10	3.77	6	No MS^2^	[M-H]^−^
PT 22	C_36_H_26_O_18_	746.11	745.10	5.58	6	No MS^2^	[M-H]^−^
PT 23	C_36_H_26_O_18_	746.11	745.10	2.57	6	No MS^2^	[M-H]^−^
PT 24	C_42_H_30_O_21_	870.13	869.12	6.05	7	No MS^2^	[M-H]^−^
PT 25	C_42_H_30_O_21_	870.13	869.12	9.69	7	DDA for preferred ion	[M-H]^−^
PT 26	C_42_H_30_O_21_	870.13	869.12	12.02	7	No MS^2^	[M-H]^−^
PT 27	C_42_H_30_O_21_	870.13	869.12	9.39	7	DDA for preferred ion	[M-H]^−^
PT 28	C_42_H_30_O_21_	870.13	869.12	6.96	7	No MS^2^	[M-H]^−^
PT 29	C_48_H_34_O_24_	994.14	993.13	11.07	8	DDA for preferred ion	[M-H]^−^
PT 30	C_48_H_34_O_24_	994.14	993.14	12.37	8	DDA for preferred ion	[M-H]^−^
PT 31	C_48_H_34_O_24_	994.14	993.14	10.30	8	No MS^2^	[M-H]^−^
PT 32	C_48_H_34_O_24_	994.14	993.14	2.54	8	No MS^2^	[M-H]^−^
PT 33	C_54_H_38_O_27_	1118.16	1117.15	13.14	9	No MS^2^	[M-H]^−^

**Table 3:** Oligonucleotide sequences of primers used for gene expression analysis in *C. elegans*.

Gene	Forward Primer Sequence (5′ → 3′)	Reverse Primer Sequence (5′ → 3′)	Accession Number
**Reference genes**			
***act-1***	GTGTTCCCATCCATTGTC	GCTCATTGTAGAAGGTGTG	NM_073418.5
***ama-1***	CTCCGTCGTTGACTGTAT	ATACCCATTCCTCGTCTTC	NM_068122.6
***pmp-3***	ATACGAAGCCACGGATAG	CTGTGTCAATGTCGTGAAG	NM_001269679.1
**Target genes**			
***daf-2***	GTGAGGACGAGCTACTATAC	TACGGGCTCACTTCATTC	AF012437
***daf-16***	GAATGGATGGTCCAGAATG	GATTCCTTCCTGGCTTTG	AF032112.1
***gcs-1***	GATTCCCAGGTCTCATTTC	GCAGGATGAGATTGTACG	NM_063526.6
***gpx-5***	CGCTGGAGTCAATGTAAAG	GGGAAGGCAATGAGAGTA	NM_077214
***gst-4***	GTGCCTTACGAGGATTATAG	GTGATAGACATTGACTGACC	NM_069447
***mtl-1***	CAAGTGCGGAGACAAATG	AGTTCCCTGGTGTTGATG	NM_072295
***pmk-1***	CATTGTTCCCTGGATCTG	CTTGAGGAGTTGCTTGAG	NM_068964
***prdx-2***	GACACCAACCACCAAATC	CTTCTCGACGAACTGGAA	NM_001383831
***skn-1***	GCAAGAGATGCGTGATTC	GTAGGCGTAGTTGGATGT	NM_171345.4
***sod-3***	GTGGTGGACACATCAATC	GCAATATCCCAACCATCC	NM_078363
***sod-4***	GGAGATACTGGAAATGGTTG	CACTTAATGAGGCAAGAGAG	NM_001268074.2

## Data Availability

All data obtained during this study are available from the corresponding author on reasonable request.

## Supplementary Materials

The following supporting information can be downloaded at https://www.mdpi.com/article/10.3390/md23080322/s1, Figure S1: AN and FV do not have toxic effects on Caco-2, IPEC-J2 cells, and *C. elegans* at the tested doses; Figure S2: AN and FV do not alter antioxidant gene expression in PQ-stressed *C. elegans*.
