# Supplementary material for: Antioxidant Power of Brown Algae: Ascophyllum nodosum and Fucus vesiculosus Extracts Mitigate Oxidative Stress In Vitro and In Vivo
Source: Mar Drugs. 2025 Aug 6;23(8):322. doi: 10.3390/md23080322 (PMC12387151; doi:10.3390/md23080322)
Supplement: Supplementary file 1 [file marinedrugs-23-00322-s001.zip › marinedrugs-3711161-supplementary.pdf]

marine drugs

Article

Antioxidant power of brown algae: *Ascophyllum nodosum* and *Fucus vesiculosus* extracts mitigate oxidative stress *in-vitro* and *in-vivo*

Lea Karlsberger [1,2], Georg Sandner [2], Lenka Molčanová [3], Tomáš Rýpar [4], Stephanie Ladirat [5], and Julian Weghuber [1,2,*]

[1] Center of Excellence Food Technology and Nutrition, University of Applied Sciences Upper Austria, Stelzhamerstraße 23, 4600 Wels, Austria; lea.karlsberger@fh-wels.at
[2] FFoQSI GmbH, Austrian Competence Centre for Feed and Food Quality, Safety and Innovation, Technopark 1D, 3430 Tulln, Austria; georg.sandner@ffoqsi.at
[3] Department of Natural Drugs, Faculty of Pharmacy, Masaryk University, Palackého třída 1946/1, 612 00 Brno, Czech Republic; molcanoval@pharm.muni.cz
[4] Department of Chemistry and Biochemistry, Mendel University in Brno, Zemědělská 1665/1, 613 00 Brno, Czech Republic; tomas.rypar@gnj.cz
[5] NUQO S.A.S, 13 Rue d'Albigny, 74000 Annecy, France; ladirat.stephanie@nuqo.eu
* Correspondence: julian.weghuber@fh-wels.at

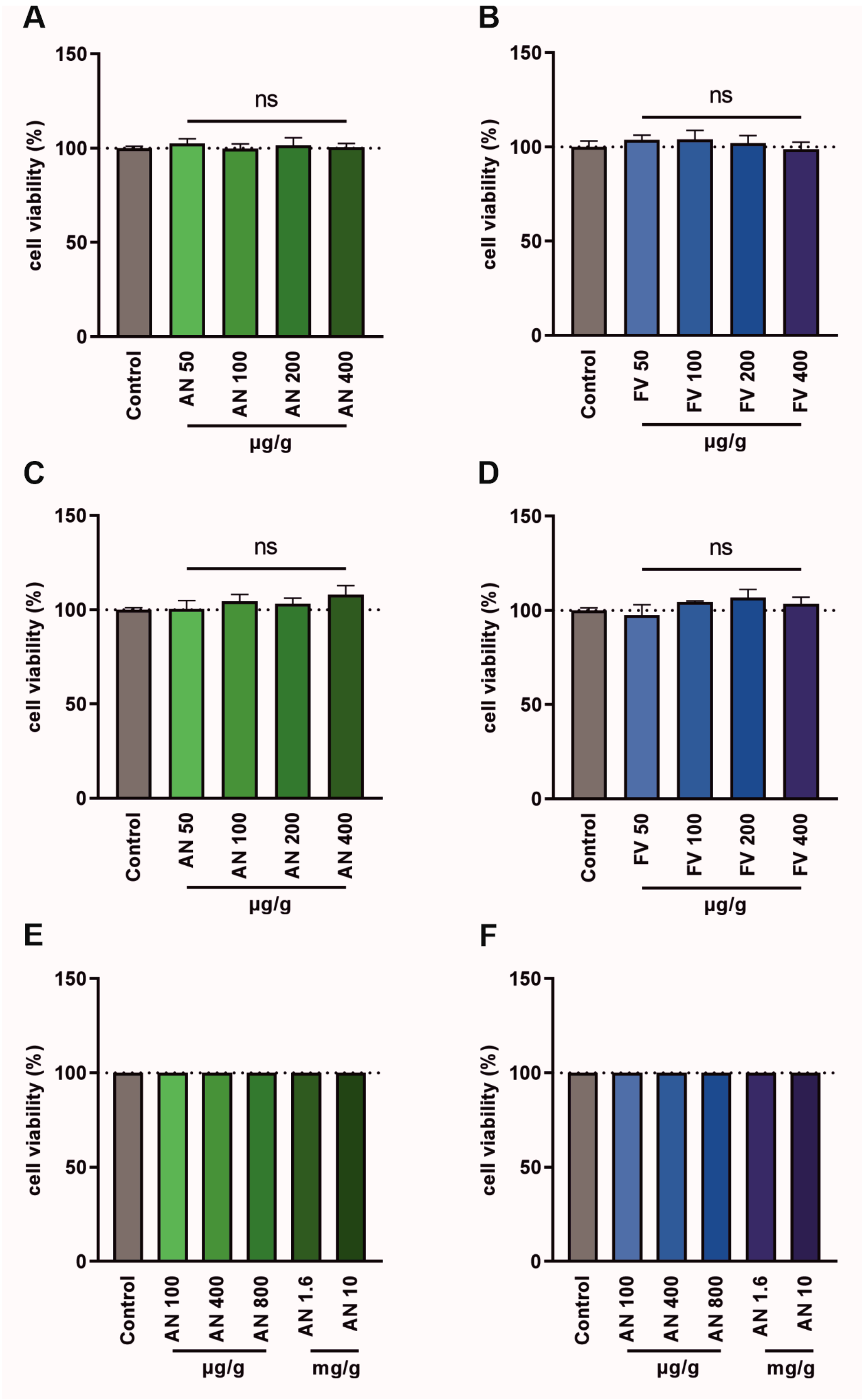

Figure S1. **AN and FV do not have toxic effects on Caco-2, IPEC-J2 cells and *C. elegans* at the tested doses.** (**A**,**B**) Human Caco-2 and (**C**,**D**) porcine IPEC-J2 cells were seeded into 96-wells plates at a density of 5 × 10⁴ or 2 × 10⁴ cells per well, respectively and grown overnight. Cells were treated with 0, 50, 100, 200 and 400 μg/g (**A**,**C**) AN or (**B**,**D**) FV for 24 h. Cell viability was measured using a resazurin-based *in-vitro* toxicology assay. Cell viability was normalized to the untreated control cells. Age-synchronized wild-type nematodes were incubated on NGM agar containing 0, 100, 400, 800 μg/g and 1.6 and 10 mg/g (**E**) AN or (**F**) FV for 72 h. The numbers of live and dead worms were determined by visual inspection. Survival rates were calculated as the percentage of live worms relative to the total number assessed. Statistical analysis was performed using one-way ANOVA followed by Dunnett's multiple comparison test. Data are shown as mean ± SD of three replicates per group and experimental day. ns = not significant.

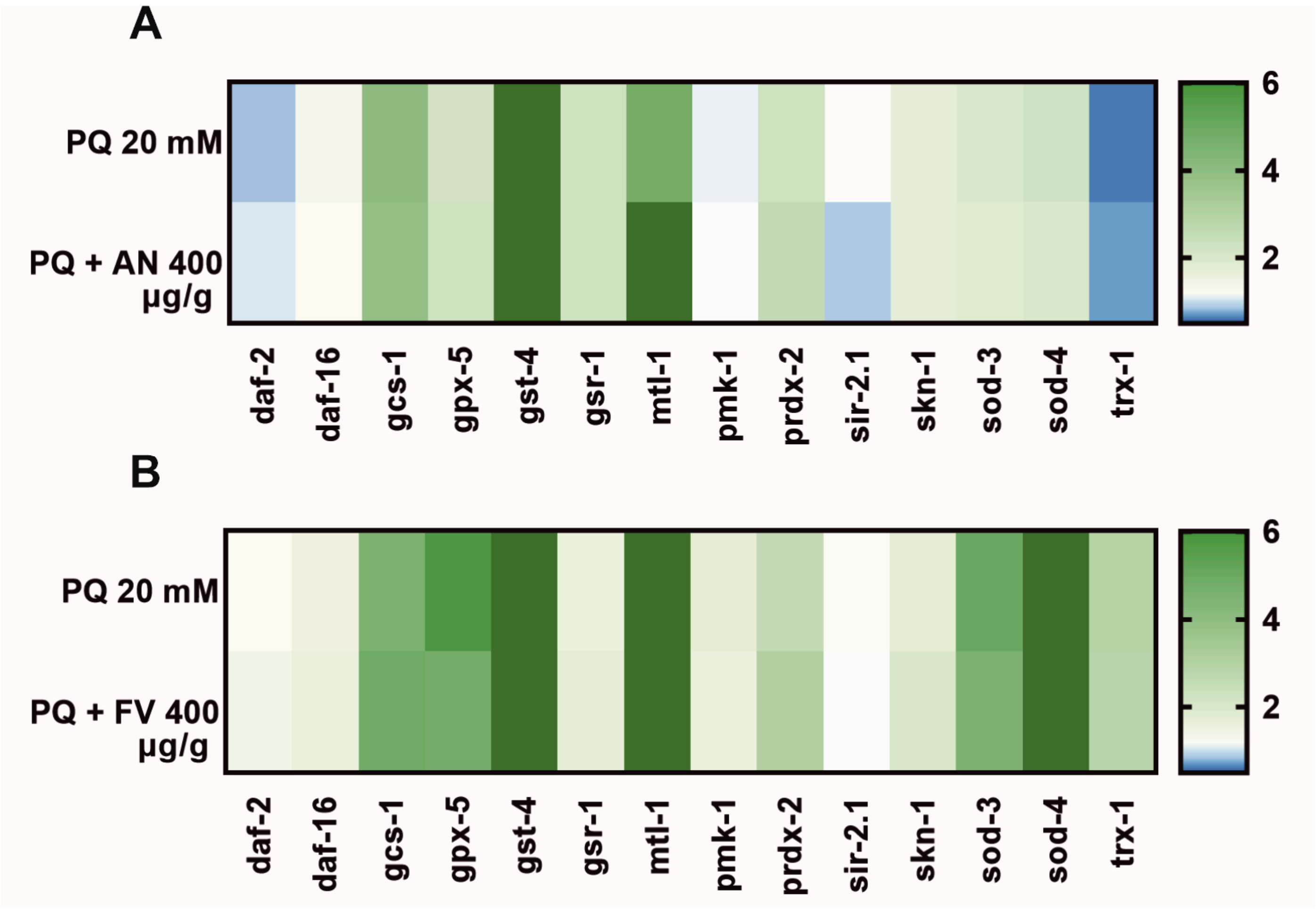

Figure S2. AN and FV do not alter antioxidant gene expression in PQ-stressed *C. elegans*. Age-synchronized wild-type nematodes were incubated on NGM agar containing 0 or 400 µg/g AN or FV for 48 h. Nematodes were transferred to NGM plates containing 20 mM PQ or control plates and incubated for additional 24 h. Gene expression was quantitated by RT-qPCR and analyzed using the $2^{-\Delta\Delta Ct}$ method. Differences compared to PQ stress controls were evaluated by one-way ANOVA with Dunnett's multiple comparison test. Changes in gene expression following (**A**) AN and (**B**) FV treatment are summarized in respective heatmaps with data shown as mean values from two independent experiments with three technical replicates per condition.
